# Antibodies targeting the glycan cap of Ebola virus glycoprotein are potent inducers of the complement system

**DOI:** 10.1038/s42003-024-06556-0

**Published:** 2024-07-17

**Authors:** Philipp A. Ilinykh, Kai Huang, Bronwyn M. Gunn, Natalia A. Kuzmina, Kritika Kedarinath, Eduardo Jurado-Cobena, Fuchun Zhou, Chandru Subramani, Matthew A. Hyde, Jalene V. Velazquez, Lauren E. Williamson, Pavlo Gilchuk, Robert H. Carnahan, Galit Alter, James E. Crowe, Alexander Bukreyev

**Affiliations:** 1 Department of Pathology, University of Texas Medical Branch, Galveston, TX USA; 2 Galveston National Laboratory, Galveston, TX USA; 3 Ragon Institute of MGH, MIT and Harvard, Cambridge, MA USA; 4 Paul G. Allen School of Global Health, Washington State University, Pullman, WA USA; 5 Vanderbilt Vaccine Center, Vanderbilt University Medical Center, Nashville, TN USA; 6 Department of Pediatrics, Vanderbilt University Medical Center, Nashville, TN USA; 7 Department of Pathology, Microbiology, and Immunology, Vanderbilt University Medical Center, Nashville, TN USA; 8 Department of Microbiology and Immunology, University of Texas Medical Branch, Galveston, TX USA

**Keywords:** Antibodies, Ebola virus, Complement cascade

## Abstract

Antibodies to Ebola virus glycoprotein (EBOV GP) represent an important correlate of the vaccine efficiency and infection survival. Both neutralization and some of the Fc-mediated effects are known to contribute the protection conferred by antibodies of various epitope specificities. At the same time, the role of the complement system remains unclear. Here, we compare complement activation by two groups of representative monoclonal antibodies (mAbs) interacting with the glycan cap (GC) or the membrane-proximal external region (MPER) of GP. Binding of GC-specific mAbs to GP induces complement-dependent cytotoxicity (CDC) in the GP-expressing cell line via C3 deposition on GP in contrast to MPER-specific mAbs. In the mouse model of EBOV infection, depletion of the complement system leads to an impairment of protection exerted by one of the GC-specific, but not MPER-specific mAbs. Our data suggest that activation of the complement system represents an important mechanism of antiviral protection by GC antibodies.

## Abstract (web summary)

An analysis of the complement activation by human monoclonal antibodies to Ebola virus glycoprotein suggests the distinct mechanisms for antibodies targeting the glycan cap versus antibodies against the membrane-proximal external region.

## Introduction

Filoviruses include one of the deadliest human pathogens known to date. *Ebolavirus* genus of the *Filoviridae* family includes Ebola virus (EBOV), Sudan virus (SUDV), Bundibugyo virus (BDBV), Taï Forest virus (TAFV), Reston virus (RESTV), and Bombali virus (BOMV)^1^. EBOV, SUDV, and BDBV are known to cause outbreaks and epidemics of highly lethal disease, which is often accompanied by hemorrhagic manifestations and systemic multiorgan dysfunction, with unpredictable periodicity, location, and scale^2^. The largest known ebolavirus epidemic took place in 2013–2016 in West Africa and was caused by EBOV. It claimed the lives of 11,310 out of 28,616 people infected^3^.

Currently, monoclonal antibody (mAb) therapy has been shown to be the most effective treatment of filoviral infections after the onset of symptoms^4^. In 2020, two mAb-based therapeutics were developed and approved by the Food and Drug Administration for clinical use^5,6^ Notably, however, these therapeutics are only effective against EBOV but not other ebolaviruses. Therefore, efficacious treatments against other pathogenic filovirus species are urgently needed.

Ebolavirus glycoprotein (GP) is the sole envelope viral protein responsible for cell entry and, hence, serves as the primary target for antibody-based therapies and vaccine design efforts. EBOV GP precursor is a 676-residue, type I transmembrane protein. It is cleaved by the host subtilisin-like proprotein convertase furin in the Golgi into two subunits, GP1 and GP2, which remain associated through a disulfide bond^7^. The GP1/GP2 heterodimer assembles into a 450 kDa trimer at the surface of nascent virions. The larger GP1 subunit encompasses the glycan cap (GC), mucin-like domain (MLD), and receptor-binding site (RBS). It is believed that the heavily glycosylated GC and MLD participate in immune evasion by restricting the antibody access to GP1 core, including the RBS^8–10^. The GP2 subunit contains the hydrophobic internal fusion loop (IFL), two heptad repeats (HR1 and HR2), membrane-proximal external region (MPER) and transmembrane anchor^8^. After attachment to a cell membrane via low-affinity interactions, virions enter the cells by macropinocytosis mechanism^11^. At low pH inside endosomes, the cathepsins B and L cleave GP to remove GC and MLD, revealing RBS for the interaction with specific filovirus receptor, the Niemann-Pick C1 (NPC1) protein^12,13^. This interaction triggers the fusion between viral and host membranes and release of the nucleocapsids into the cytoplasm.

GC-targeting mAbs alone are protective in animal challenge models and likely contribute to overall protection during natural infections^14–16^ and for vaccine-mediated protection of challenged animals^17^. Although neutralizing activity is considered to be the major mechanism of protection by mAbs^18^, there is increasing evidence that Fc-effector function contributes to the control and clearance of filoviral infections^19–25^, and even neutralizing mAbs may require Fc functions to confer optimal levels of protection^26^. Several therapeutic antibody combinations, including ZMapp^27^, REGN-EB3^28^, FVM04/CA45^29^, MBP134^AF^^30^, rEBOV-520/548^31^, rEBOV-442/515^32^, and 1C3/1C11^33^, were generated and shown to be protective in the non-human primate challenge models. GC-specific antibodies are important components of the most of these combinations and are known to form a large portion of the humoral immune response to natural ebolavirus infection^34–36^. Since GC is dispensable for virus entry, antiviral mechanisms employed by mAbs targeting GC remain unclear, although a recent study suggests that GC-specific mAbs can indirectly inhibit GP proteolysis by shifting the MLD position and sterically occluding the cathepsin cleavage loop^37^. Other mechanisms independent of Fab-mediated virus neutralization have been proposed for GC-specific mAbs, including antibody-dependent cellular phagocytosis (ADCP) and activation of NK cells^18,23^.

Complement is a host defense system comprising more than 30 soluble protein factors and cell surface receptors in blood and other body fluids that interact to sense and respond to invading pathogens. This system can be activated through the classical, alternative, or lectin pathway, but only the classical pathway is antigen- and antibody-dependent, thus bridging the innate and adaptive immune systems. The classical pathway is triggered by binding of C1q to the Fc domain of antigen-bound antibodies (typically, IgG1, IgG3, or IgM). The C1q molecule is an assembly of six heterotrimers, each with a binding site for Fc. In plasma, C1q forms a complex with C1r and C1s serine proteases. Once C1q has bound multiple Fc regions, C1r and C1s get activated resulting in cleavage of the complement proteins C4 and C2 into larger (C4b, C2a) and smaller (C4a, C2b) fragments. The larger fragments associate to form the C3 convertase, C4bC2a, which cleaves C3 into C3a and C3b. The latter binds covalently to reactive surfaces and “label” pathogens and infected cells for subsequent elimination via phagocytosis. C3b also interacts with C4bC2a complex forming the C5 convertase, C4bC2aC3b, which cleaves C5 into C5a and C5b. C5b is deposited onto the activating surface and initiates irreversible binding of C6, C7, C8, and multiple copies of C9 to form the membrane attack complex (MAC). MAC permeates the lipid bilayer, causing the lysis of antigen-expressing cells or enveloped viral particles^38–40^.

Several studies suggested that the complement system is an important component of EBOV neutralization by antibodies during the natural infection^41^ and immunization of experimental animals^42,43^. Moreover, a recent Fc-engineering study illustrated the possibility of tuning the mAb protective potential in vivo by introduction of mutations regulating the activation of complement. Specifically, it was shown that KWES set of amino acid mutations in an Fc fragment of VIC16 mAb, which upregulates complement activation, improves the protection of mice from EBOV infection compared to unmodified mAb^25^. However, the direct requirement of complement for mAb-mediated protection against filovirus infections has not been demonstrated.

In the present study, we compared complement activation by ebolavirus GP-specific mAbs with different epitope specificities. First, using an antibody-dependent complement deposition (ADCD) assay, GC-specific mAbs were shown to better induce C3 deposition compared to MPER mAbs. Second, we developed a complement-dependent cytotoxicity (CDC) assay and demonstrated that GC-specific mAbs stimulate killing of the target antigen-expressing cells by complement, an activity that can be inhibited by mAbs recognizing the other parts of GP, such as the MPER or base region. Using the chemical inhibitor of N-linked glycosylation, we further showed that N-linked glycans on the GP surface, while serving as part of the mAb epitope, can nevertheless downregulate mAb-mediated CDC activity. This finding represents a previously unknown mechanism of evasion of antiviral complement activity employed by EBOV. Finally, the depletion of complement in mice by injection of the cobra venom factor (CVF) impaired the survival of EBOV-challenged animals upon treatment with a GC-specific mAb, but not the MPER mAbs, indicating requirement of the functional complement system for effective protection by at least some of the GC-specific mAbs. These results contribute to understanding the mechanisms of virus-complement interplay and highlight an important role of the complement system in anti-EBOV activity. The obtained data can inform the selection of GC-specific mAbs for improved therapeutic antibody combinations.

## Results

### Glycan cap mAbs are more potent complement activators compared to MPER mAbs

From our previously published studies on human mAbs isolated from survivors of ebolavirus infection^26,34,37,44–47^, we selected a panel of neutralizing GC- or MPER-specific mAbs to determine a possible difference in complement activation between these two groups of antibodies. A well-characterized 13C6 mouse mAb^42^, which is a component of MB-003 and ZMapp combinations against EBOV and known to neutralize virus only in the presence of complement^20,27^, and mAbs ADI-15820, ADI-16061^35,36^ and KZ52^48^ isolated from human survivors of EBOV infection, also were included in this panel. For some antibodies, the recombinant versions bearing L234A/L235A/P329G (LALA-PG) or K322A (KA) mutation in the Fc fragment were produced as controls. The LALA-PG set of mutations in the Fc region is one of the most effective at silencing Fc-mediated activity^49^, and the KA mutation greatly reduces binding to C1q resulting in the lack of efficient activation of complement^50^.

**Fig. 1:**
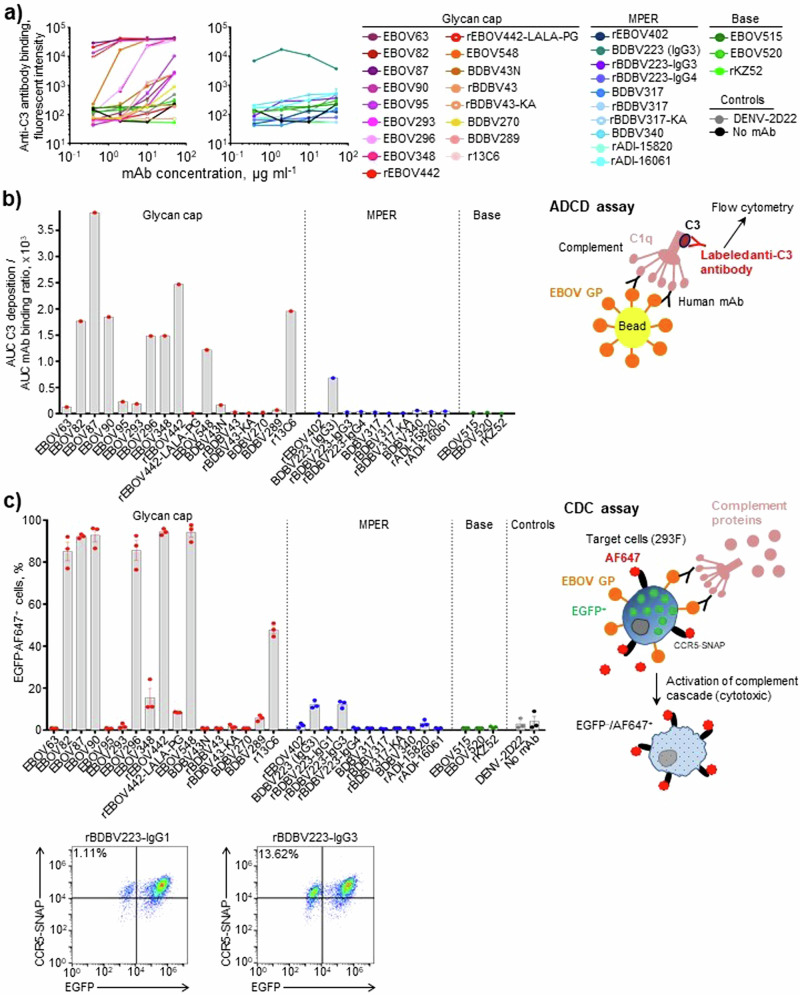
Glycan cap mAbs activate complement more potently than MPER mAbs. Antibody-dependent complement deposition (ADCD) assay. **a** Dose–response curves. **b** Relative C3 deposition (normalized to mAb binding with beads). GP-coated beads were incubated with mAbs and complement, and mAb binding and C3 deposition were quantified by flow cytometry. The results were expressed as a ratio of area under the curve values for C3 deposition and mAb binding to beads. **c** Complement-dependent cytotoxicity (CDC) assay. 293F cells expressing EGFP, EBOV GP, and the chimeric CCR5-SNAP tag protein were labeled with AF647 and incubated with mAbs (10 µg ml^−1^) and complement (10%). The cytotoxicity was determined as percentage of EGFP^–^AF647^+^ cells by flow cytometry. (**b**) and (**c**) also include schematic representations of ADCD or CDC assays, respectively. Representative dot plots are shown. **a**, **c**: mean ± SEM of triplicate samples are shown.

First, we measured a dose-dependent mAb ability to induce C3 deposition onto GP-coated beads in ADCD assay (Fig. 1a, b). All the mAbs tested, except for rBDBV223-IgG3 and -IgG4, belonged to the IgG1 subclass, allowing us to dissect the role of the epitope specificity in the activity. High levels of C3 deposition were observed for the GC-specific mAbs, whereas most of the tested mAbs specific to MPER, EBOV GP base region, or irrelevant target mAb (DENV-2D22; specific to dengue virus envelope protein^51^) did not show activity. We next asked whether the observed mAb effects on C3 deposition translated into the complement-mediated killing of antigen-expressing cells. For this work, we developed a CDC assay using the EBOV GPkik-293FS EGFP CCR5-SNAP cell line^52^. This cell line constitutively expresses EBOV GP on the plasma membrane, EGFP in the cytoplasm and the SNAP-tag CCR5, which can be specifically labeled with SNAP-Surface Alexa Fluor 647, on the cell surface. Fluorophore-labeled cells were incubated consecutively with the mAbs and complement, and the cytotoxicity was quantified as a percentage of EGFP^–^AF647^+^ cells^53,54^ by analytical flow cytometry (Fig. 1c). CDC activity was observed for nearly half of the GC-specific mAbs tested, and with the hybridoma-produced and recombinant (rBDBV223-IgG3) versions of BDBV223 mAb, both belonging to the IgG3 subclass. The results are consistent with the data in Fig. 1a, b, except for rBDBV223-IgG3, which did not show activity in the ADCD assay. This finding potentially could be attributed to differences in mAb binding to the bead-conjugated versus membrane-displayed GP in ADCD or CDC assays, respectively.

**Fig. 2:**
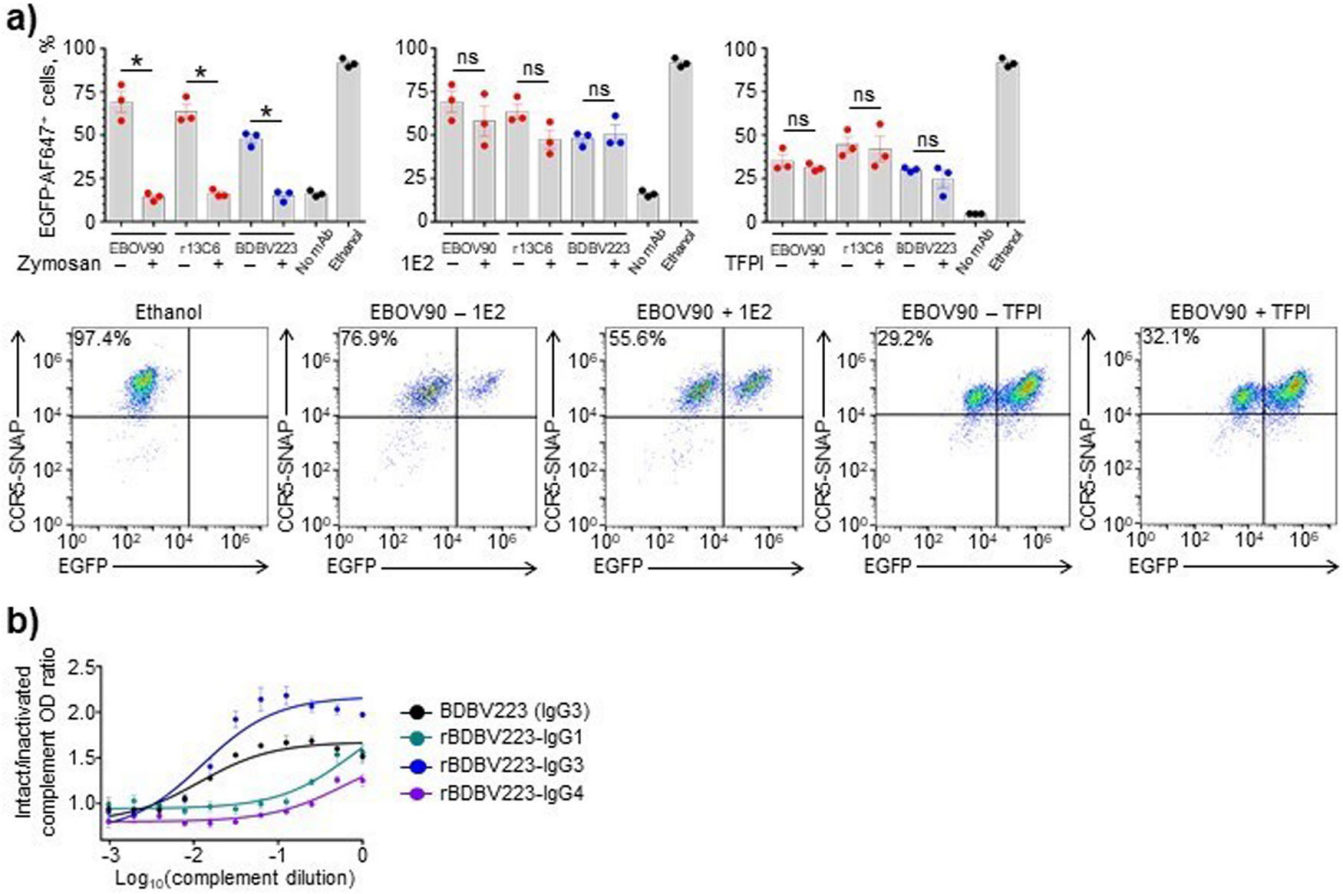
Intact complement is required for CDC and ADCD mAb activity. **a** Specificity of CDC assay. Complement was pre-treated with zymosan at 20 mg ml^−1^ (left) or 1E2 antibody at 0.1 mg ml^−1^ (middle) and added to cells, or 293F cells were treated with 1 µg ml^−1^ TFPI and incubated with complement (right). As controls, cells were mock-treated (no mAb), or treated with absolute ethanol (cell death control). *p < 0.001; ns, not significant (unpaired t-test). Representative dot plots are shown. **b** Difference in complement activation by BDBV223 isotypes (10 µg ml^−1^) validated by ELISA. Antigen-bound mAbs were incubated with serially diluted intact or heat-inactivated complement and the results were expressed as a ratio of C3-specific OD signals (OD intact compl./OD heat-inact. compl.). Mean ± SEM of triplicate samples are shown.

The specificity of the CDC assay was further validated in separate experiments using selected mAbs with high CDC activities. When complement was pre-treated with zymosan A, which was expected to consume the complement system activity^55^, the cytotoxicity was significantly reduced for all antibodies regardless of the epitope specificity or IgG subclass (Fig. 2a, left). These results suggest that the cell killing activities observed for mAbs in the developed assay depend specifically on the presence of intact complement. Conversely, when complement was pre-treated with antibody 1E2 against the mannose-binding lectin (MBL), changes in mAb activity were not detected (Fig. 2a, middle). These data also were confirmed when tissue factor pathway inhibitor (TFPI) was added to cells along with the mAbs (Fig. 2a, right). TFPI is known as a selective inhibitor of MASP-2 serine protease of the lectin pathway, which does not affect the classical pathway proteases C1s or C1r^56^. Altogether, our data demonstrate that the observed mAb-driven cytotoxicity results from activation of the classical complement pathway.

IgG subclass-specific differences observed in ADCD and CDC assays were confirmed by an ELISA method for hybridoma-derived (IgG3) and recombinant (IgG1, IgG3, IgG4) versions of BDBV223 (Fig. 2b). In concordance with Fig. 1a–c data, the C3 deposition activity of BDBV223 mAb subclasses decreased in the following order: IgG3 > IgG1 > IgG4.

### MPER- and base-region-specific mAbs block cytotoxicity induced by GC or MPER mAbs

**Fig. 3:**
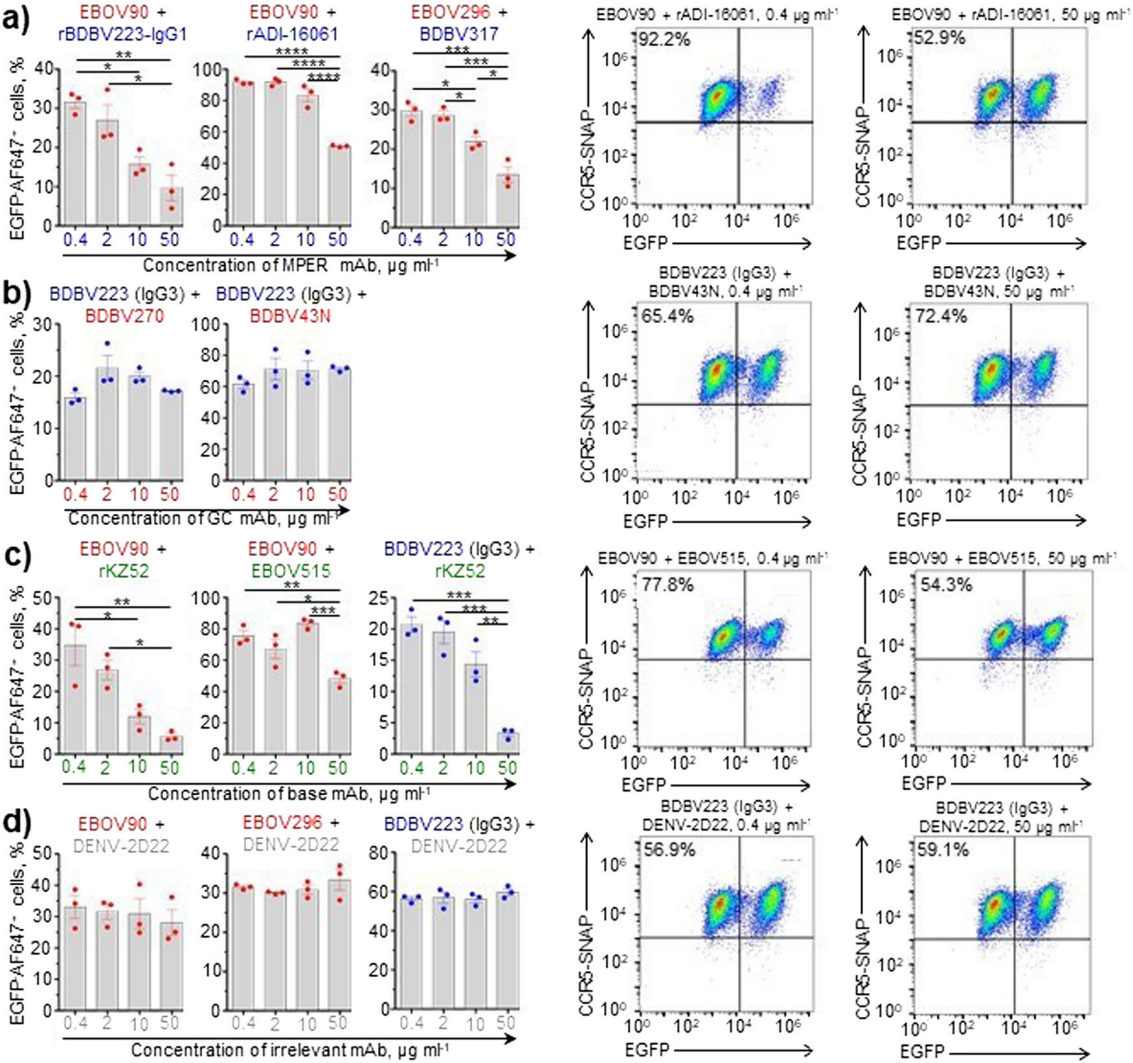
MPER and base mAbs block cytotoxicity induced by either GC or MPER mAbs. CDC assay with pairs of mAbs of various epitope specificities (**a**–**d**). In each pair, mAb indicated first was used at 10 µg ml^−1^, and mAb indicated second was used at increasing concentrations from 0.4 to 50 µg ml^−1^ (color-matched). **a** GC/MPER pairs, **b** MPER/GC pairs, **c** GC/base and MPER/base pairs, **d** controls – GC and MPER mAbs paired with an irrelevant DENV-2D22 mAb. Mean ± SEM of triplicate samples are shown. *p < 0.05; **p < 0.01; ***p < 0.001; ****p < 0.0001 (ANOVA, Tukey’s multiple comparisons test). Representative dot plots are shown.

We next tested if antibodies of various epitope specificities could interact to block each other’s CDC activities. The following combinations were tested: high-activity GC mAbs with low-activity MPER mAbs (Fig. 3a); high-activity MPER mAb with low-activity GC mAbs (Fig. 3b); and high-activity GC or MPER mAbs with low-activity base mAbs (Fig. 3c). As expected, addition of an irrelevant isotype-control antibody DENV-2D22 did not change the activity of tested mAbs, regardless of their epitopes (Fig. 3d), highlighting the specificity of our assay. The results of these experiments show that: (1) MPER-specific mAbs can dose-dependently inhibit CDC activity of the GC-specific mAbs, but not vice versa; and (2) base-region-specific mAbs can dose-dependently inhibit the activity induced by both GC- and MPER-specific mAbs.

**Fig. 4:**
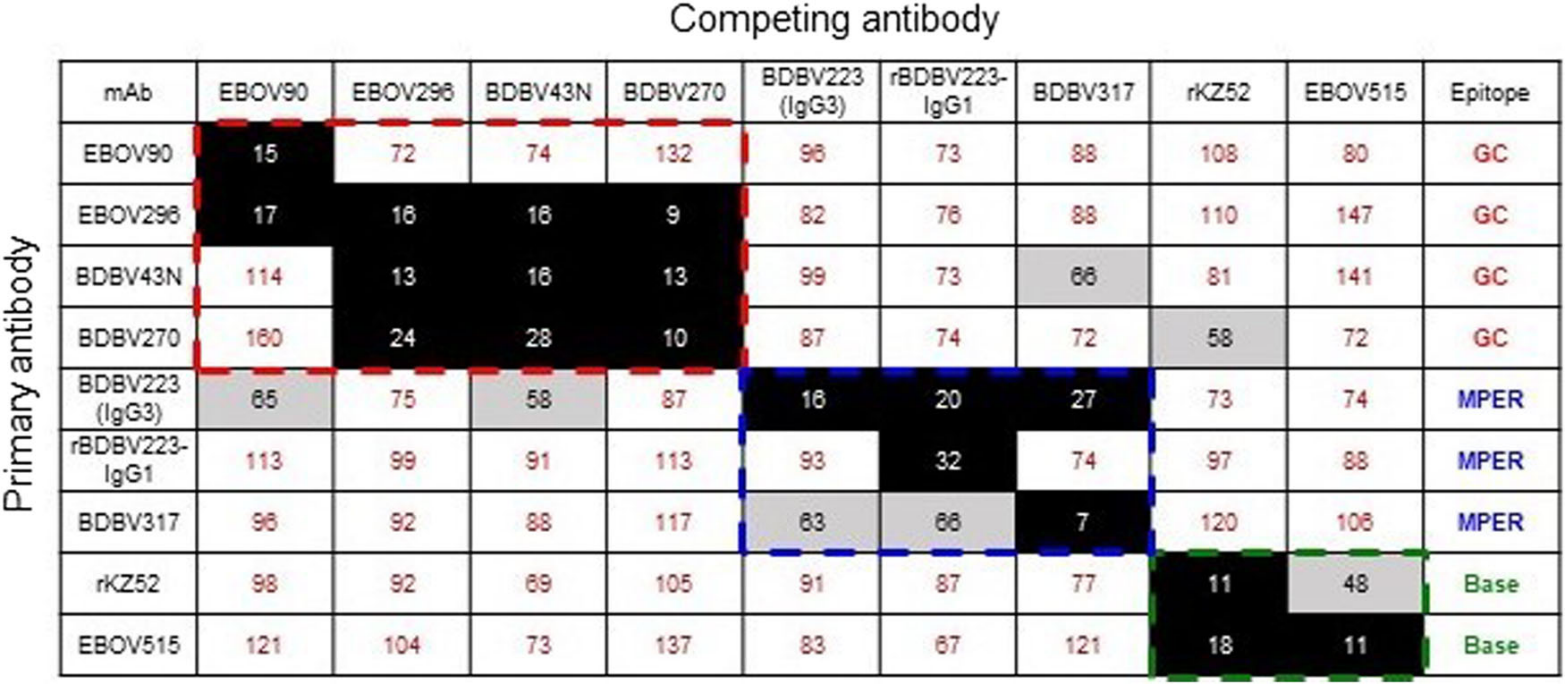
GC, MPER, and base mAbs have non-overlapping epitopes. Competition-binding ELISA assay. Numbers indicate the percent binding of the competing mAb in the presence of the first mAb, compared to binding of competing mAb in the presence of an irrelevant negative control mAb, DENV-2D22. Full, intermediate, or no competition was defined based on the reduction in percent binding to <33% (black boxes with white numbers), 33 to 66% (gray boxes with black numbers), or >66% (white boxes with red numbers), respectively. The assay was repeated three times with comparable results.

Theoretically, the data obtained could result from the scenario when binding of low-activity mAbs simply prevents the subsequent binding of highly active mAbs to EBOV GP. To explore this possibility, we performed an ELISA-based competition-binding analysis to see how binding of one mAb affects binding of a second mAb with a different epitope specificity (Fig. 4). As expected, all mAbs clustered according to their antigenic site specificity (GC, MPER or base region), suggesting that the observed inhibition of CDC activity of GC or MPER mAbs by inactive MPER or base mAbs was unlikely to have resulted from direct mAb competition for their EBOV GP target.

### N-linked glycans on EBOV GP prevent CDC

**Fig. 5:**
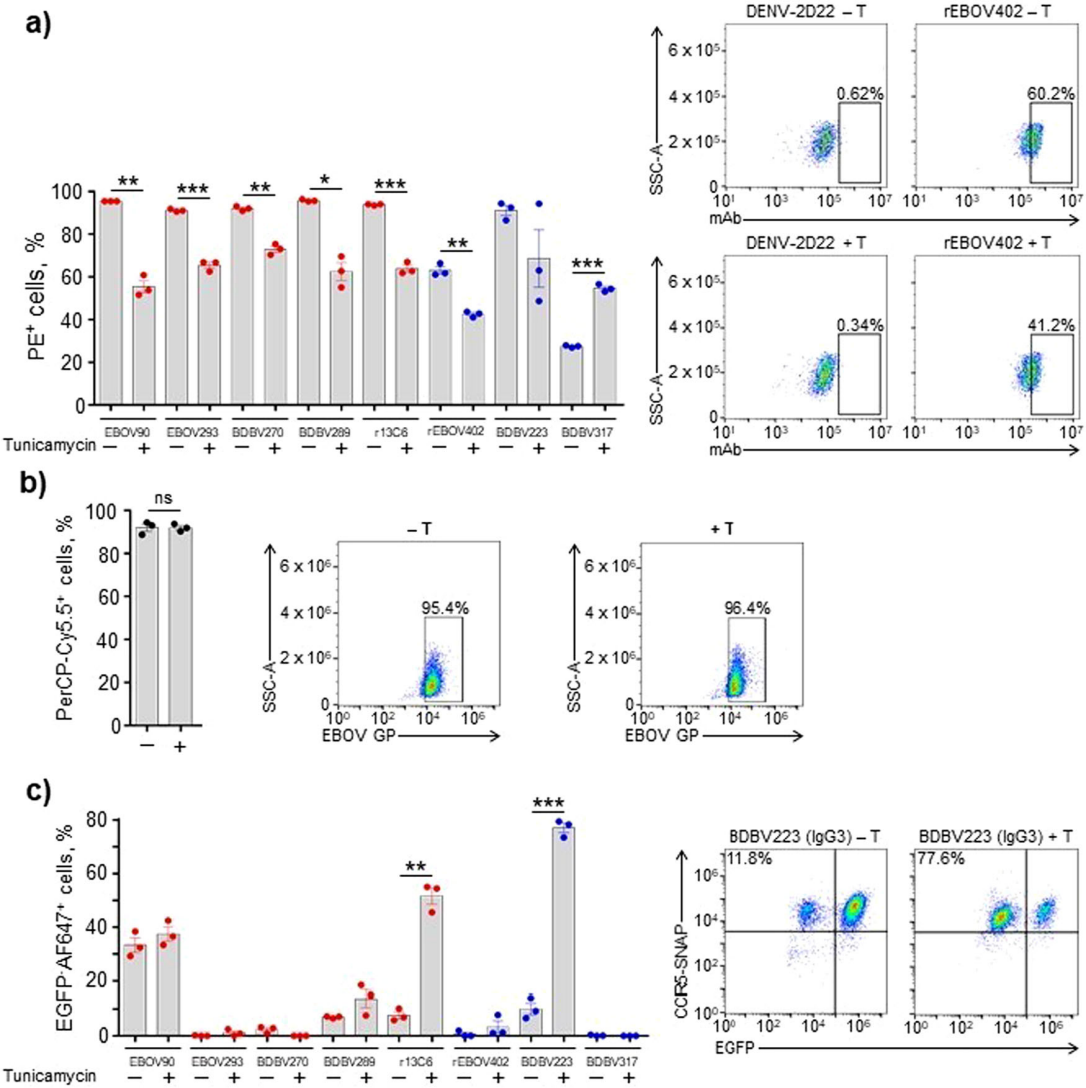
N-linked glycans on EBOV GP prevent activation of CDC mechanism. **a** SNAP-tagged 293F cells expressing EGFP and EBOV GP were treated with the vehicle control (–) or 1 µg ml^−1^ tunicamycin (+) and incubated with 10 µg ml^−1^ mAbs. Binding of mAbs to EBOV GP was determined by flow cytometry using PE-conjugated goat anti-human IgG secondary antibody. **b** SNAP-tagged 293F cells were treated as in (**a**), and the surface expression of EBOV GP was determined by flow cytometry using rabbit anti-EBOV VLP antiserum and mouse anti-rabbit IgG secondary antibody conjugated with PerCP-Cy5.5. **c** Cells were treated as in (**a**), and CDC assay was performed as in Fig. 1c. The percentages of EGFP^–^AF647^+^ cells in samples treated with the vehicle control or tunicamycin and incubated with DENV-2D22 mAb were used for background signal subtraction. Mean ± SEM of triplicate samples are shown. *p < 0.01; **p < 0.001; ***p < 0.0001; ns, not significant (unpaired t-test). Representative flow cytometry dot plots are shown.

EBOV GP is a heavily glycosylated protein^8,57^, which can affect multiple biological properties of the virus^58^. We next explored a possible role of N-glycans on EBOV GP in modulating mAb-induced CDC activity. First, we tested if pre-treatment of cells with tunicamycin, a chemical inhibitor of N-linked glycosylation^59^, would alter mAb binding to EBOV GP. Tunicamycin treatment resulted in a significant decrease of mAb binding to 293F cells expressing EBOV GP, except for the BDBV317 MPER-specific mAb (Fig. 5a). At the same time, this treatment did not have a detectable effect on GP expression on the surface of target cells (Fig. 5b), suggesting that an impairment of mAb binding was not due to a reduction of GP expression level caused by tunicamycin. Interestingly, when the CDC assay was run for mAbs following tunicamycin treatment of cells, the opposite effect was observed: an increase, rather than decrease of activity, for some of the tested mAbs, regardless of the epitope specificity (Fig. 5c). Therefore, even though N-deglycosylation of GP disfavors mAb binding, it nevertheless results in hyperactivation of the complement-mediated lysis of target cells induced by mAbs. Overall, these data show that N-linked glycans on EBOV GP protect cells from CDC.

### Some GC-specific, but not MPER-specific mAbs, require complement for in vivo protection against EBOV

**Fig. 6:**
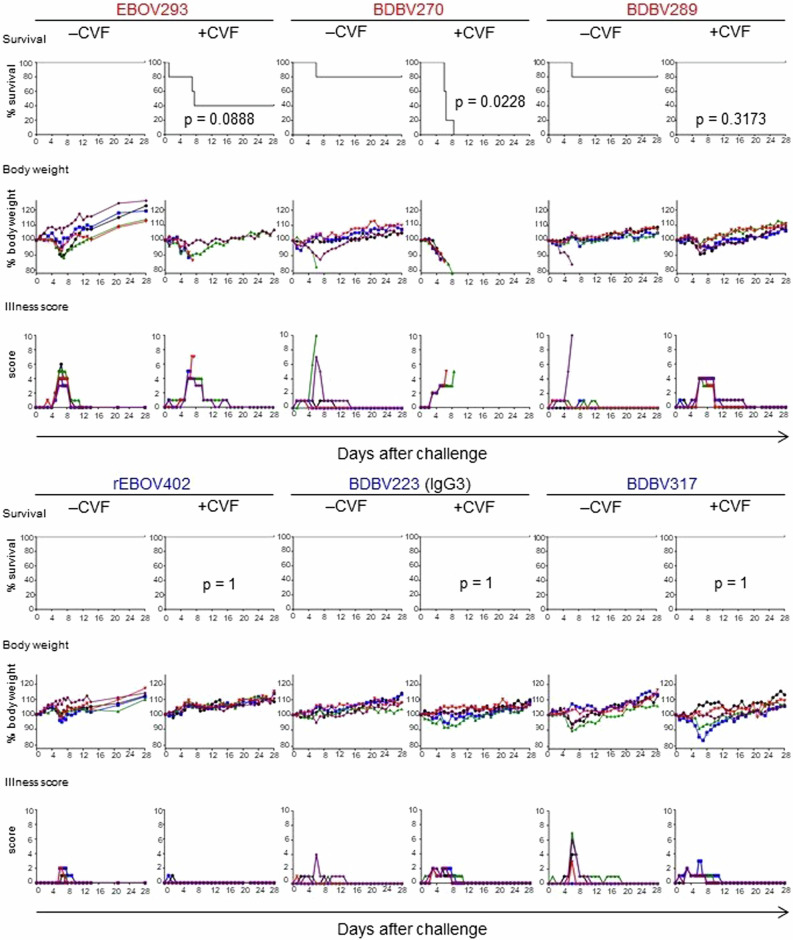
Some glycan cap mAbs require complement for in vivo protection against EBOV. Groups of mice at five animals per group were injected with indicated mAbs by the IP route at 24 h after EBOV challenge. Additionally, mice were treated with PBS (–CVF) or CVF (+CVF) one day prior to infection and at 3 dpi. Kaplan–Meier survival curves, body weight, and illness score curves are shown. For each mAb, –CVF and +CVF groups are compared (Mantel-Cox test). In EBOV293 + CVF group, one mouse succumbed at 1 dpi and was therefore excluded from the analysis.

Finally, we addressed the role of the complement system in mAb-mediated protection against EBOV in vivo. Groups of BALB/c mice were treated with CVF to deplete their complement system, or mock-treated and next day exposed to a lethal (1000 PFU) dose of mouse-adapted EBOV. On day 1 after infection mice were treated with individual mAbs at 100 μg (∼5 mg kg^−1^) or mock-treated, and on day 3 after infection, treatment or mock-treatment with CVF was repeated (Fig. 6). Notably, for one of the three tested GC-specific mAbs, BDBV270, the protection was completely abrogated by CVF, and for another, EBOV293, the protection was markedly reduced. In contrast, CVF treatment did not abrogate the protection by any of the MPER-specific mAbs tested. These data suggest that activation of the complement system is an important antiviral mechanism, which is required for in vivo protection conferred by at least some of the mAbs targeting the GC, but not the MPER of EBOV GP.

## Discussion

Using a combination of in vitro and in vivo approaches, we investigated the role of the complement system in antiviral mechanisms employed by antibodies directed to EBOV GP. First, using a bead-based ADCD assay, we compared the ability of two mAb groups with different epitope specificities to induce the C3 deposition on GP-coated surface. GC-specific mAbs were shown to be superior to their MPER-specific counterparts in ADCD activity (Fig. 1a, b). These data are in line with the results of a previous study, which analyzed multiple functional activities for 168 EBOV GP mAbs. Notably, antibodies targeting the most exposed GP regions, such as the head, GC, and MLD, demonstrated stronger engagement of Fc-effector functions compared to mAbs against the conformationally obscured, “hidden” epitopes (i.e., HR2/MPER, IFL)^18,23^. This observation was hypothesized to result from a greater accessibility of Fc fragments of mAbs bound to outer GP regions for the interaction with Fc receptors at the surface of immune cells, or with the complement system components^60^.

Deposition of complement can lead to formation of MAC and lysis of lipid membranes of enveloped viruses^61^ or infected host cells expressing viral antigens^62^. Complement deposition on virion particles may contribute to direct elimination of viral particles but probably is not critical for protection by mAbs that neutralize virus without complement^34,37^. Elimination of infected cells by complement-enhanced mechanisms, however, is more likely to reduce total viral burden. To test if the observed mAb ability to induce C3 deposition would result in an increased mAb-mediated cytotoxicity, we developed a CDC assay using a human-origin (human embryonic kidney 293F) cell line constitutively expressing EBOV GP^52^ (Fig. 1c). The relative activity of individual mAbs in the CDC assay was similar to that determined by the ADCD assay, confirming the functional relevance of C3 deposition. High activity also was detected for an IgG3 form of the BDBV223 MPER mAb, the only antibody for which other subclasses in addition to IgG1 were tested. These results also were confirmed by ELISA (Fig. 2b). It is known that Fc-mediated activities vary greatly among IgG subclasses. The amino acid sequence of the C_H_2 region^63^ and the antibody hinge region length^64^ determines the complement-fixing potential of antibodies. IgG3 has the most potent affinity for binding to C1q, followed by IgG1, with a very weak association for IgG2 and no detectable interaction for IgG4^40,65^. From that perspective, BDBV223-IgG3 serves as a positive control in the tested panel.

The specificity of the 293F-cell-based CDC assay we developed was validated with complement-depleting or pathway-inhibiting compounds. First, using zymosan A, we showed that the mAb-mediated cytotoxicity requires the presence of intact complement. Zymosan is a carbohydrate substance extracted from yeast cell walls and is a potent activator of the alternative complement pathway. Zymosan can directly interact with properdin, the regulatory plasma glycoprotein produced by neutrophils which forms the stabilizing complex with C3bBb convertase (C3bBbP). After non-covalent attachment to the surface of zymosan particles, properdin binds C3b and initiates assembly of C3bBbP complexes, facilitating the prompt depletion of C3 complement component by the amplification convertase^66^. Second, using TFPI and anti-MBL antibody, we demonstrated that the observed CDC activity results from activation of the classical, but not lectin, complement pathway and, therefore, specifically requires the presence of GP-specific antibodies (Fig. 2a).

Next, considering the difference in complement activation by GC- and MPER-specific mAbs, we questioned the possible biological outcome of interaction between these two antibody groups, as should normally happen in a context of a polyclonal antibody response to EBOV infection and/or vaccination. We found that base-specific mAbs dose-dependently inhibited CDC activity of the GC-specific and MPER mAbs, and that MPER mAbs inhibited the activity of GC mAbs (Fig. 3). Due to the lack of competition between GC, MPER and base mAbs for the target (Fig. 4), this inhibition is unlikely to be explained by a simplistic model where binding of low-activity mAbs to GP prevents binding of highly active mAbs. It is possible that interference takes place at the later stage, following the binding of Fab fragments of mAbs from the different epitope groups to GP. For instance, the simultaneous occupation of two or more epitopes may cause the conformational GP alterations unfavorable for complement activation, *i.e*., by precluding efficient complement binding to Fc fragments due to steric hindrance. In any case, understanding the mechanisms of the observed phenomenon requires future investigation. Of note, an antibody of the IgG3 subclass (BDBV223) was used as a sole MPER mAb with CDC activity. Therefore, it would be relevant to search for an IgG1 MPER antibody able for the complement-mediated cell killing, and, should any have been identified, test it in combinations with low-activity GC and base mAbs to confirm our findings. Importantly, analysis of the convalescent plasma IgG protein repertoire in a survivor of the 2013–2016 West African EBOV epidemic identified the GC, base region and head domain/RBS as the most abundantly recognized antigenic sites on GP^67^. A longitudinal study of B cell responses to natural EBOV infection revealed the persistence of IgG1, rapid decline of IgG3, late appearance of IgG4 and the absence of IgG2 antibodies specific to the viral GP^68^. Therefore, regulation of complement activation at polyclonal antibody level is likely to be a complex process, given the overall diversity of immunogenic epitopes and the dynamics of IgG subclass composition.

Filovirus GPs are heavily glycosylated with both N-linked and O-linked glycans, and glycans contribute from one-third to one-half of their molecular weight^69^. The glycosylation of ebolavirus GP is extremely heterogeneous, with some sites carrying over 40 unique glycan compositions, and it is even possible that the virion surface does not contain two copies of GP with the exact same glycosylation pattern^70^. There are up to 17 N-linked glycosylation sites in ebolavirus GP, 5 of which are located in GC^70^. GP1 N-glycans are suggested to participate in an immune evasion by shielding the epitopes from antibody recognition^57^. The GP2 subunit contains two N-linked glycosylation sites that contribute GP expression, stability, and cell entry^71,72^. Given the important role of N-linked glycans in virus structure and life cycle, we addressed their possible effect on the mAb CDC activity using tunicamycin. Initially identified as a natural antibiotic, tunicamycin is now widely used for blocking N-linked glycosylation by inhibiting the transfer of UDP-N-acetylglucosamine to dolichol phosphate in the endoplasmic reticulum of eukaryotic cells^73–75^. For most of the tested mAbs, tunicamycin treatment reduced binding to GP (Fig. 5a). This finding was unexpected, since removal of glycans by mutagenesis was shown to enhance sensitivity of vesicular stomatitis virus (VSV) pseudotyped with EBOV GP (VSV/EBOV-GP) to neutralization by whole IgG purified from the serum of vaccinated or convalescent cynomolgus macaques^57,71^. However, our data suggest that, for certain mAbs, N-linked glycans may be a part of their epitopes, rather than shielding the epitopes from immune recognition. In particular, the epitopes for mAbs 13C6 and BDBV289 contain N238 and N268 glycans, and the EBOV293 mAb epitope contains an N268 glycan^70^. Interestingly, removal of the N563 glycan site by mutagenesis enhanced VSV/EBOV-GP neutralization for some mAbs, while impairing it for the other mAbs^36^. In our panel, the only mAb that demonstrated an increased GP binding in the presence of tunicamycin was BDBV317. Its epitope can be shielded by a N618 glycan, which is located close to the escape mutation site identified for this mAb^44^. It should be noted that tunicamycin treatment does not allow dissection of the role of specific glycans, which can be studied in part by site-directed mutagenesis approaches^36,57,71,72^. However, tunicamycin treatment has the advantage that it does not change the amino acid residue at the site of glycosylation, minimizing a possible impact on GP expression (Fig. 5b). Surprisingly, tunicamycin treatment not only did not reduce the CDC activity, as one could expect based on mAb binding data (Fig. 5a), but, instead, caused an increase of the cytotoxicity for some of the tested mAbs (Fig. 5c). To our knowledge, the phenomenon of specific downregulation of the classical complement pathway by N-linked glycans at the viral surface has not been described. A possibility is that EBOV employs GP glycosylation to reduce the antiviral complement activity.

Finally, we selected a few available well-characterized GC- and MPER-specific mAbs, for which we have previously reported protection in vivo^26,34,37,45^, and addressed the role of the complement system using CVF treatment in the mouse model of EBOV infection. CVF shares structural and functional properties with C3. It also has C3b-like activity in forming the extremely stable CVF-dependent convertase, CVF,Bb, which cleaves C3 and C5 components^76^. Treatment of BALB/c mice with CVF depletes complement^77,78^. We showed that CVF treatment significantly impaired the protection conferred by the GC-specific mAb BDBV270 and partially reduced the protection conferred by other GC-specific mAbs, but did not impair the protection by BDBV289 mAb or any MPER-specific mAb (Fig. 6). BDBV289 is more potently neutralizing compared to mAbs BDBV270 and EBOV293^34,37^, and was shown to protect in mouse, guinea pig^34^ and rhesus macaque^79^ models of ebolavirus infection. It is therefore possible that BDBV289-mediated protection relies mainly on Fab-dependent virus neutralization and does not require complement activation. Similarly, MPER-specific antibodies can protect through direct virus neutralization, likely by interfering with the viral fusion machinery^80^.

Ebolaviruses continue to pose a significant threat to public health by inducing outbreaks and epidemics of a highly lethal disease. Passive immunotherapy remains the most reliable therapeutic option for prophylaxis and post-exposure treatment. Therefore, understanding of protective mechanisms used by antibodies is critical to inform development of the most effective immunotherapeutic regimens and design of vaccines. In the present study, we addressed the antiviral mechanism for GC-binding mAbs. We showed that (1) GC mAbs are superior to MPER mAbs in complement activation; (2) CDC activity can be dose-dependently inhibited by complement-inactive mAbs of different epitope specificity; (3) N-linked glycans can serve as a part of a mAb epitope and (4) N-linked glycans greatly downregulate CDC activity. The role of the complement system in mAb-mediated protection against EBOV in vivo was tested with a limited number of mAbs. While depletion of C3 did not affect the protection by any of the three tested MPER-specific mAbs, it completely abrogated the protection by one of the three tested GC-specific mAbs, and markedly reduced the protection by another mAb. Testing of a greater number of GC-specific mAbs is required to better understand the role of C3 in vivo. Altogether, our results highlight the previously underappreciated role for activation of the complement system as an important mechanism of antibody-mediated protection against EBOV.

## Methods

### Cell lines

293F cells expressing EBOV GP (strain Kikwit) on the plasma membrane, EGFP in the cytoplasm and the SNAP-tag CCR5 on the cell surface^52^ were kindly provided by Dr. George K. Lewis (University of Maryland). The cell suspension was maintained in FreeStyle^TM^ 293 expression medium (Gibco) containing 1 µg ml^−1^ puromycin (InvivoGen) at 37 °C in 8% CO_2_ shaken at 130 rpm. Vero-E6 cells (green monkey kidney epithelial) were obtained from ATCC (CRL-1586). Cells were maintained in minimum essential medium supplemented with 10% fetal bovine serum and 1% penicillin-streptomycin solution (Gibco) at 37 °C in 5% CO_2_.

### Viruses

The mouse-adapted EBOV strain Mayinga (EBOV-MA, isolate EBOV/M.mus-tc/COD/76/Yambuku-Mayinga, GenBank accession number: AF499101) was originally generated by Dr. Mike Bray (U.S. Army Medical Research Institute of Infectious Diseases)^81^. The virus was provided originally by the Special Pathogens Branch of CDC, deposited in the World Reference Center for Emerging Viruses and Arboviruses at UTMB, and amplified by one passage in Vero-E6 cells. To determine the titer, virus was inoculated onto Vero-E6 cell culture monolayers and incubated for 14 days under 0.45% methylcellulose (Thermo Fisher Scientific) overlay. Then, monolayers were fixed with formalin (Thermo Fisher Scientific), and viral plaques were immunostained with rabbit polyclonal antibody against EBOV GP (IBT Bioservices), Horse radish peroxidase (HRP)-labeled goat anti-rabbit IgG secondary antibody (Thermo Fisher Scientific) and Vector NovaRED peroxidase substrate kit (Vector Laboratories).

### Production of hybridoma-derived and recombinant mAbs

Hybridoma mAbs EBOV63, EBOV82, EBOV87, EBOV90, EBOV95, EBOV293, EBOV296, EBOV348, BDBV270, BDBV289, BDBV317 (IgG1 isotype), and BDBV223 (IgG3 isotype) were isolated from a human survivor of a natural EBOV or BDBV infection and purified from cultured hybridoma cell supernatants as described previously^34,37^. MAb DENV-2D22 (IgG1 isotype) that is specific to dengue virus envelope (E) protein was described previously^51^. Recombinant mAbs BDBV43, BDBV270, EBOV402, BDBV223, BDBV317, rEBOV-548, rEBOV-442, rEBOV-442-LALA-PG, BDBV340, rADI-16061, rEBOV-515, rEBOV-520, and mAbs ADI-15820 and KZ52 were produced in mammalian Expi293F or ExpiCHO cells (Gibco). ADI-15820 and KZ52 were produced based on known heavy- and light-chain variable region genes for these mAbs. Murine-human chimeric mAb c13C6 was produced in Expi293F cells based on known variable region sequences of murine mAb 13C6 with human IgG1 Fc region. BDBV43N was expressed in a tobacco plant (*Nicotiana benthamiana*) and was kindly provided by Dr. Larry Zeitlin (Mapp Biopharmaceutical, Inc.). Antibody heavy- and light-chain variable region genes were sequenced from hybridoma lines that had been cloned biologically by flow cytometric sorting. Briefly, total RNA was extracted using the RNeasy Mini kit (QIAGEN) and reverse-transcriptase PCR (RT-PCR) amplification of the antibody gene cDNAs was performed using the PrimeScript One Step RT-PCR kit (Takara Bio Inc.) according to the manufacturer’s protocol with gene-specific primers^82^. The thermal cycling conditions were as follows: 50 °C for 30 min, 94 °C for 2 min, 40 cycles of (94 °C for 30 s, 58 °C for 30 s and 72 °C for 1 min). PCR products were purified using Agencourt AMPure XP magnetic beads (Beckman Coulter) and sequenced directly using an ABI3700 automated DNA analyzer. For recombinant mAb production, cDNA encoding the genes of heavy and light chains were cloned into DNA plasmid monocistronic expression vectors for mammalian cell culture mAb secretion encoding IgG1, IgG3, IgG4, or IgG1-KA heavy chain^83^ and transformed into *Escherichia coli* cells. This vector contains an enhanced 2A sequence and GSG linker that enables simultaneous expression of mAb heavy- and light-chain genes from a single construct after transfection. MAb proteins were produced following transiently transfection of Expi293F or ExpiCHO cells following the manufacturer’s protocol and were purified from filtered culture supernatants by fast protein liquid chromatography on an ÄKTA instrument using HiTrap MabSelect Sure or HiTrap Protein G columns (GE Healthcare). Purified mAbs were buffer exchanged into phosphate buffered saline (PBS), filtered using sterile 0.45-μm pore size filter devices (Millipore), concentrated, and stored in aliquots at −80 °C until use. Purification of hybridoma-produced mAbs is described elsewhere^84^.

### Analysis of mAb IgG subclass specificity

The isotype and subclass of secreted antibodies were confirmed by ELISA using murine anti-human IgG1, IgG3, or IgG4 mouse antibodies conjugated with alkaline phosphatase (Southern Biotech).

### Measurement of mAb binding to GP-covered beads

Biotinylated recombinant EBOV GP (IBT Biotherapeutics) was coupled to red fluorescent Neutravidin beads (Life Technologies). Monoclonal antibodies were diluted in PBS in an 8-point 5-fold dilution curve, starting from 50 µg ml^−1^. Fifty microliters per well of antibodies were incubated with GP-coupled beads for 2 h at 37 °C in a 96-well plate, and each antibody concentration was assayed in duplicates. Beads were pelleted at 1000 × *g* for 10 min to remove unbound antibody. Antibody-coated beads were then incubated with an APC-conjugated anti-human IgG antibody (Biolegend) at a 1:200 dilution in PBS for 15 min at room temperature. Beads were washed twice with PBS by centrifugation at 1000 × *g* for 5 min and fixed with 4% paraformaldehyde for 15 min at room temperature before washing and final resuspension with PBS. The MFI of APC on red neutravidin beads was determined using a Cytek Aurora Spectral flow cytometer and SpectroFlo software. Data were normalized to a positive control on each plate, and duplicates were averaged.

### Antibody-mediated complement deposition (ADCD)

Recombinant EBOV GP with the transmembrane domain removed (GPΔTM) (Mayinga strain; IBT Bioservices) was biotinylated using LC-LC-Sulfo-NHS Biotin (Thermo Fisher Scientific). Excess biotin was removed using a Zeba desalting column (Thermo Fisher Scientific). Biotinylated GP antigen was then coupled to 1 µm red Neutravidin beads (Thermo Fisher Scientific) by incubating beads and antigen overnight at 4 °C. Beads were washed twice with PBS containing 0.1% bovine serum albumin (BSA). mAbs were diluted in unsupplemented RPMI1640 (Gibco) and incubated with GP-coated beads for 2 h at 37 °C. Unbound antibodies were removed by centrifugation prior to the addition of reconstituted guinea pig complement (Cedarlane Labs) diluted in veronal buffer supplemented with calcium and magnesium (Boston Bioproducts) for 20 min at 37 °C. Beads were washed with PBS containing 15 mM EDTA and stained with an Fluorescein-5-isothiocyanate (FITC)-conjugated anti-guinea pig C3 antibody (MP Biomedicals). C3 deposition onto beads was measured using a BD LSRII flow cytometer (BD Biosciences). The geometric mean fluorescent intensity of FITC of all beads was measured. Data analysis was performed using FlowJo (BD Biosciences) Version X.

### C3c-specific ELISA

Flat-bottom high-binding 96-well microplates (Greiner Bio-One) were coated at 4 °C overnight with purified EBOV GP (Mayinga strain; Sino Biologicals) diluted at 1 µg ml^−1^ in PBS and washed four times with PBST buffer (0.1% Tween-20 in PBS). Bound antigen was blocked with 0.5% bovine serum albumin (BSA; Sigma-Aldrich) in PBST buffer for 30 min at room temperature. Then, blocking buffer was removed, and mAbs were added in triplicates at 10 μg ml^−1^ in PBST-0.5% BSA and the plates were incubated for 1 h at room temperature. Plates were washed four times in PBST, two-fold serial dilutions of human complement sera (Sigma-Aldrich) in PBST-0.5% BSA from 1:1 to 1:2048 were added, and plates were incubated for 20 min at 37 °C. Dilutions of heat-inactivated complement (30 min, 56 °C) were added to control wells. After four washes with PBST, HRP-conjugated sheep anti-human C3c secondary antibody (Thermo Fisher Scientific) diluted at 1:500 in blocking buffer was added, and plates were incubated for 1 h at room temperature. Next, plates were washed four times in PBST, KPL SureBlue TMB peroxidase substrate solution (SeraCare) was added, and plates were incubated for 10 min at room temperature. The reaction was stopped by an equal volume of KPL TMB BLUESTOP solution (SeraCare), and plates were scanned in a Synergy microplate reader (BioTek) at the emission wavelength 630 nm. The results were expressed as a ratio of C3-specific OD signals after incubation of antigen-bound mAbs with serially diluted intact or heat-inactivated complement.

### Complement-dependent cytotoxicity (CDC) assay

SNAP-tagged 293F cells expressing EGFP and EBOV GP (0.5 × 10^6^ cells per sample) were washed with PBS containing 1% BSA and incubated for 30 min with SNAP-Surface Alexa Fluor (AF) 647 substrate (New England BioLabs) at 37 °C, 8% CO_2_, 130 rpm. Then, cells were washed three times with PBS and incubated in triplicates at room temperature with indicated concentrations of mAb or mAb mixtures diluted in FreeStyle^TM^ 293 expression medium. In 15 min, baby rabbit complement (Cedarlane) was added up to a final concentration of 10%, and cells were incubated on a shaker for 6 h at 37 °C, 8% CO_2_, 130 rpm, washed twice with PBS-1% BSA, fixed with 4% methanol-free formaldehyde solution (Thermo Fisher Scientific) and kept overnight at 4 °C in dark. Next, cells were washed twice with PBS and analyzed by flow cytometry using an Accuri C6 cytometer (BD Biosciences). The cytotoxicity of the mAb was determined as the percentage of cells losing EGFP (by virtue of CDC) but retaining the surface expression of CCR5-SNAP (EGFP^–^AF647^+^).

In some experiments, the complement was pre-treated with 20 mg ml^−1^ zymosan A (Sigma-Aldrich) or 0.1 mg ml^−1^ 1E2 antibody (Abcam) for 1 h at 37 °C before addition to cells, or TFPI (Sigma-Aldrich) was added to cells together with antibodies up to the final concentration of 1 µg ml^−1^. Absolute ethanol was used as a cell death control.

In N-deglycosylation experiments, cells were treated overnight on a shaker at 37 °C, 8% CO_2_, 130 rpm with 1 µg ml^−1^ tunicamycin (Sigma-Aldrich) diluted in ethanol, or treated with 0.1% ethanol (vehicle control), and then subjected to CDC assay. Same concentrations of tunicamycin or its diluent were maintained during incubation of cells with mAbs and complement. The percentages of EGFP^–^AF647^+^ cells in samples treated with the vehicle control or tunicamycin and incubated with DEBV-2D22 mAb were used for background signal subtraction.

### EBOV GPΔTM expression and purification

For competition-binding studies, the ectodomain of EBOV GPΔTM (residues 1–636; strain Makona; GenBank: KM233070) containing a C-terminal strep II tag was expressed and purified as previously described^85^.

### Biotinylation of mAbs

For competition-binding studies, mAbs were biotinylated using the EZ-LinkTM NHS-PEG4-Biotin, No-WeighTM Format (Thermo Fisher Scientific) at room temperature for 30 min. To remove residual biotin, the mAbs were buffer exchanged into PBS using 7–40 K MWCO Zeba spin desalting columns (Thermo Fisher Scientific).

### Competition-binding analysis via ELISA

Wells of 384-well plates (Thermo Fisher Scientific) were coated with EBOV GPΔTM protein diluted in PBS and incubated at 4 °C overnight. The plates were aspirated and blocked for 1 h at room temperature with blocking solution (2% BSA and 0.05% Tween-20 in PBS). After blocking, the plates then were washed three times with 0.05% Tween-20 in PBS and the first Ab (20 µg ml^−1^) in blocking solution (1% BSA and 0.05% Tween-20 in PBS) was added at 20 µl per well for 1 h at room temperature. Biotinylated second Ab (5 µg ml^−1^; final concentration of 1 µg ml^−1^) was added in blocking solution (1% BSA and 0.05% Tween-20 in PBS) at 5 µl per well for 1 h at room temperature. The plates then were washed three times with 0.05% Tween-20 in PBS and incubated with a solution of secondary Abs (mouse anti-biotin-HRP, Southern Biotech) diluted 1:4000 in blocking solution (1% BSA and 0.05% Tween-20 in PBS) for 1 h at room temperature. The plates were washed three times with 0.05% Tween-20 in PBS followed by addition of One-step Ultra-TMB ELISA substrate solution (Thermo Fisher Scientific). The reaction was stopped with 1 N HCl and then read at an optical density of 450 nm with a BiotekTM plate reader. Percent binding of each Ab was normalized to the optical density value for binding in the presence of an irrelevant negative control mAb, DENV-2D22^51^. Full, intermediate, or no competition was defined based on the reduction in percent binding to <33%, 33 to 66%, or >66%, respectively. The assay was repeated three times with essentially the same results.

### MAb binding to tunicamycin-treated 293F cells

Cells were treated overnight with tunicamycin or vehicle control as described above, washed twice with PBS-1% BSA, and incubated in triplicates with 10 µg ml^−1^ mAbs diluted in PBS-1% BSA for 20 min at room temperature. Then, cells were washed twice with PBS-1% BSA and incubated with PE-conjugated goat anti-human IgG secondary antibody (Thermo Fisher Scientific) diluted at 1:200 in PBS-1% BSA for 20 min in dark at room temperature. After two washes with PBS-1% BSA, cells were fixed with 4% formaldehyde and kept overnight at 4 °C in dark. Next, cells were washed twice with PBS, and the percentages of antibody-bound cells (PE^+^) were determined by flow cytometry as above. The percentages of PE^+^ cells in samples treated with the vehicle control or tunicamycin and incubated with DENV-2D22 mAb were used for background signal subtraction.

### GP expression on tunicamycin-treated cells

Cells were treated overnight with tunicamycin or vehicle control as described above, washed twice with PBS-1% BSA, and incubated in triplicates with rabbit anti-EBOV VLP antiserum (IBT Bioservices) diluted at 1:100 in PBS-1% BSA for 20 min at room temperature. Then, cells were washed twice with PBS-1% BSA and incubated with PerCP-Cy5.5-conjugated mouse anti-rabbit IgG secondary antibody (Santa Cruz Biotechnology) diluted at 1:200 in PBS-1% BSA for 20 min in dark at room temperature. After two washes with PBS-1% BSA, cells were fixed with 4% formaldehyde and kept overnight at 4 °C in dark. Next, cells were washed twice with PBS, and percentages of GP-expressing cells (PerCP-Cy5.5^+^) were determined by flow cytometry as above.

### Mouse studies

Mice were housed in microisolator cages and provided food and water ad libitum. Groups of 7–8-week-old BALB/c mice (Charles River Laboratories) were inoculated with 1000 PFU of the EBOV-MA by the intraperitoneal (i.p.) route in 100 µl PBS. Viral inoculate was back titrated at time of infection to verify viral titer. Mice (*n* = 5) were treated i.p. with 20 µg (or approximately 1 unit) of CVF (Sigma-Aldrich) in 500 µl PBS or mock-treated at one day prior to and three days after the challenge, and with 100 μg (~5 mg kg^−1^) of individual mAb in 100 µl PBS on day 1 post-challenge. Mice were monitored twice daily from day 0 to day 14 post-challenge for illness, survival, and weight loss, followed by once daily monitoring from day 15 to the end of the study at day 28, as described elsewhere^86^. Moribund mice were euthanized as per the approved protocol (see Ethics statement). All mice were euthanized on day 28 after EBOV challenge.

### Statistics and reproducibility

Statistical analyses and generation of graphs were performed using GraphPad Prism version 6.07 (GraphPad Software). One-way ANOVA with multiple comparisons (Tukey’s test) or a t-test were used for statistical data analysis. Animal survival data were analyzed by log-rank (Mantel-Cox) test. Each in vitro experiment has been conducted at least three times. The n numbers are indicated in figure legends, where appropriate. For in vivo data presented in Fig. 6, number of animals per group is indicated.

### Ethics statement

We have complied with all relevant ethical regulations for animal use. Challenge studies were conducted under maximum containment in an animal biosafety level 4 facility of the Galveston National Laboratory, UTMB. The animal protocol was approved by the Institutional Animal Care and Use Committee (protocol №1508050) in compliance with the Animal Welfare Act and other applicable federal statutes and regulations relating to animals and experiments involving animals.

### Reporting summary

Further information on research design is available in the Nature Portfolio Reporting Summary linked to this article.

### Supplementary information

Description of Additional Supplementary Materials

Supplementary datasheet 1

Reporting Summary

## Data Availability

All primary data generated or analyzed in this study are available on request from the authors. Source data behind the figures can be found in Supplementary Data sheet 1.
