# Author Correction: Antibodies targeting the glycan cap of Ebola virus glycoprotein are potent inducers of the complement system

**DOI:** 10.1038/s42003-024-06610-x

**Published:** 2024-07-31

**Authors:** Philipp A. Ilinykh, Kai Huang, Bronwyn M. Gunn, Natalia A. Kuzmina, Kritika Kedarinath, Eduardo Jurado-Cobena, Fuchun Zhou, Chandru Subramani, Matthew A. Hyde, Jalene V. Velazquez, Lauren E. Williamson, Pavlo Gilchuk, Robert H. Carnahan, Galit Alter, James E. Crowe, Alexander Bukreyev

**Affiliations:** 1 Department of Pathology, University of Texas Medical Branch, Galveston, TX USA; 2 Galveston National Laboratory, Galveston, TX USA; 3 Ragon Institute of MGH, MIT and Harvard, Cambridge, MA USA; 4 Paul G. Allen School of Global Health, Washington State University, Pullman, WA USA; 5 Vanderbilt Vaccine Center, Vanderbilt University Medical Center, Nashville, TN USA; 6 Department of Pediatrics, Vanderbilt University Medical Center, Nashville, TN USA; 7 Department of Pathology, Microbiology, and Immunology, Vanderbilt University Medical Center, Nashville, TN USA; 8 Department of Microbiology and Immunology, University of Texas Medical Branch, Galveston, TX USA

**Keywords:** Antibodies, Ebola virus, Complement cascade

Correction to: *Communications Biology* 10.1038/s42003-024-06556-0; published online 17 July 2024

In this article, the title was incorrectly given as “Antibodies targeting the glycan cap of Ebola virus glycoprotein are potent inducers of the complement“ but should have been “Antibodies targeting the glycan cap of Ebola virus glycoprotein are potent inducers of the complement system”. The original article has been corrected.

