# Supplementary material for: Antibodies targeting the glycan cap of Ebola virus glycoprotein are potent inducers of the complement system
Source: Commun Biol. 2024 Jul 17;7:871. doi: 10.1038/s42003-024-06556-0 (PMC11255267; doi:10.1038/s42003-024-06556-0)
Supplement: Supplementary file 1 — Description of Additional Supplementary Materials [file 42003_2024_6556_MOESM1_ESM.pdf]

Description of Additional Supplementary Files

File name: Supplementary Datasheet 1

Description: Source data behind the figures
