# Supplementary material for: Antibodies targeting the glycan cap of Ebola virus glycoprotein are potent inducers of the complement system
Source: Commun Biol. 2024 Jul 17;7:871. doi: 10.1038/s42003-024-06556-0 (PMC11255267; doi:10.1038/s42003-024-06556-0)
Supplement: Supplementary file 3 — Reporting Summary [file 42003_2024_6556_MOESM3_ESM.pdf]

nature portfolio

Corresponding author(s): Galit Alter, James E. Crowe, Alexander Bukreyev

Last updated by author(s): 2023-20-04

Reporting Summary

Nature Portfolio wishes to improve the reproducibility of the work that we publish. This form provides structure for consistency and transparency in reporting. For further information on Nature Portfolio policies, see our Editorial Policies and the Editorial Policy Checklist.

Statistics

For all statistical analyses, confirm that the following items are present in the figure legend, table legend, main text, or Methods section.

n/a	Confirmed	
☐	☒	The exact sample size (n) for each experimental group/condition, given as a discrete number and unit of measurement
☒	☐	A statement on whether measurements were taken from distinct samples or whether the same sample was measured repeatedly
☐	☒	The statistical test(s) used AND whether they are one- or two-sided<br>*Only common tests should be described solely by name; describe more complex techniques in the Methods section.*
☒	☐	A description of all covariates tested
☒	☐	A description of any assumptions or corrections, such as tests of normality and adjustment for multiple comparisons
☒	☐	A full description of the statistical parameters including central tendency (e.g. means) or other basic estimates (e.g. regression coefficient) AND variation (e.g. standard deviation) or associated estimates of uncertainty (e.g. confidence intervals)
☐	☒	For null hypothesis testing, the test statistic (e.g. F, t, r) with confidence intervals, effect sizes, degrees of freedom and P value noted<br>*Give P values as exact values whenever suitable.*
☒	☐	For Bayesian analysis, information on the choice of priors and Markov chain Monte Carlo settings
☒	☐	For hierarchical and complex designs, identification of the appropriate level for tests and full reporting of outcomes
☒	☐	Estimates of effect sizes (e.g. Cohen's d, Pearson's r), indicating how they were calculated

Our web collection on statistics for biologists contains articles on many of the points above.

Software and code

Policy information about availability of computer code

Data collection	n/a
Data analysis	n/a

For manuscripts utilizing custom algorithms or software that are central to the research but not yet described in published literature, software must be made available to editors and reviewers. We strongly encourage code deposition in a community repository (e.g. GitHub). See the Nature Portfolio guidelines for submitting code & software for further information.

Data

Policy information about availability of data

All manuscripts must include a data availability statement. This statement should provide the following information, where applicable:

- Accession codes, unique identifiers, or web links for publicly available datasets
- A description of any restrictions on data availability
- For clinical datasets or third party data, please ensure that the statement adheres to our policy

The datasets generated during the current study are available from the corresponding author on reasonable request.

Research involving human participants, their data, or biological material

Policy information about studies with human participants or human data. See also policy information about sex, gender (identity/presentation), and sexual orientation and race, ethnicity and racism.

Reporting on sex and gender	n/a
Reporting on race, ethnicity, or other socially relevant groupings	n/a
Population characteristics	n/a
Recruitment	n/a
Ethics oversight	n/a

Note that full information on the approval of the study protocol must also be provided in the manuscript.

Field-specific reporting

Please select the one below that is the best fit for your research. If you are not sure, read the appropriate sections before making your selection.

☒ Life sciences ☐ Behavioural & social sciences ☐ Ecological, evolutionary & environmental sciences

For a reference copy of the document with all sections, see nature.com/documents/nr-reporting-summary-flat.pdf

Life sciences study design

All studies must disclose on these points even when the disclosure is negative.

Sample size	3 – 5
Data exclusions	n/a
Replication	n/a
Randomization	n/a
Blinding	n/a

Reporting for specific materials, systems and methods

We require information from authors about some types of materials, experimental systems and methods used in many studies. Here, indicate whether each material, system or method listed is relevant to your study. If you are not sure if a list item applies to your research, read the appropriate section before selecting a response.

Materials & experimental systems

n/a	Involved in the study	
☐	☒	Antibodies
☐	☒	Eukaryotic cell lines
☒	☐	Palaeontology and archaeology
☐	☒	Animals and other organisms
☒	☐	Clinical data
☒	☐	Dual use research of concern
☒	☐	Plants

Methods

n/a	Involved in the study	
☒	☐	ChIP-seq
☐	☒	Flow cytometry
☒	☐	MRI-based neuroimaging

Antibodies

Antibodies used	• Human monoclonal antibodies isolated from survivors of ebolavirus infection (Ilinykh PA, PLoS Pathog 2018, 14(8):e1007204, PMID30138408; Flyak AI, Cell 2016, 164(3):392-405, PMID26806128; Murin CD, Cell Rep 2021, 35(2):108984, PMID33852862; Kuzmina NA, Cell Rep 2018, 24(7):1802-1815.e5, PMID30110637; Flyak AI, Nat Microbiol 2018, 3(6):670-677, PMID29736037; King LB, Nat Commun 2019, 10(1):1788, PMID30996276)

- 13C6 (Wilson JA, Science 2000, 287(5458):1664-6, PMID10698744)
- ADI-15820 (Bornholdt ZA, Science 2016, 351(6277):1078-83, PMID26912366; Wec AZ, Cell 2017, 169(5):878-890.e15, PMID28525755)
- KZ52 (Maruyama T, J Virol 1999, 73(7):6024-30, PMID10364354)
- 2D22 (Fibriansah G, Science 2015, 349(6243):88-91, PMID26138979)
- 1E2, anti-MBL antibody (Abcam, Cat. #ab23458, Lot #GR212269-1)
- Rabbit polyclonal antibody against EBOV GP (IBT Bioservices, Cat. #0301-015, Lot #1501003)
- Horse radish peroxidase (HRP)-labeled goat anti-rabbit IgG secondary antibody (Thermo Fisher Scientific, Cat. #A16110, Lot #69-130-020320)
- HRP-conjugated sheep anti-human C3c secondary antibody (Thermo Fisher Scientific, Cat. #PA1-86651)
- PE-conjugated goat anti-human IgG secondary antibody (Thermo Fisher Scientific, Cat. #PA1-86078, Lot #SG2416609A)
- Rabbit anti-EBOV VLP antiserum (IBT Bioservices, Cat. #01-0004, Lot #1302001)
- PerCP-Cy5.5-conjugated mouse anti-rabbit IgG secondary antibody (Santa Cruz Biotechnology, Cat. #sc-45109, Lot #L1217)
- Fluorescein-5-isothiocyanate (FITC)-conjugated goat anti-guinea pig C3 antibody (MP Biomedicals, Cat. #0855385, Lot #04549)

Validation

n/a

Eukaryotic cell lines

Policy information about cell lines and Sex and Gender in Research

Cell line source(s)

- Vero-E6 (ATCC: CRL-1586)
- 293F cells expressing EBOV GP (strain Kikwit) on the plasma membrane, EGFP in the cytoplasm and the SNAP-tag CCR5 on the cell surface (Domi A, Sci Rep 2018, 8(1):864, PMID29339750) were provided by Dr. George K. Lewis (University of Maryland)

Authentication

None of the cell lines used were authenticated by any method other than microscopic observation

Mycoplasma contamination

All cell lines tested negative for mycoplasma contamination

Commonly misidentified lines
(See ICLAC register)

n/a

Animals and other research organisms

Policy information about studies involving animals; ARRIVE guidelines recommended for reporting animal research, and Sex and Gender in Research

Laboratory animals

Species: Mice
Strain: BALB/c
Age: 7-8 weeks
Vendor: Charles River Laboratories

Wild animals

n/a

Reporting on sex

Female

Field-collected samples

n/a

Ethics oversight

The animal protocol for testing of human monoclonal antibodies against EBOV in cobra venom factor-treated and untreated mice was approved by the Institutional Animal Care and Use Committee (IACUC) of the University of Texas Medical Branch in compliance with the Animal Welfare Act and other applicable federal statutes and regulations relating to animals and experiments involving animals.

Note that full information on the approval of the study protocol must also be provided in the manuscript.

Flow Cytometry

Plots

Confirm that:

☒ The axis labels state the marker and fluorochrome used (e.g. CD4-FITC).

☒ The axis scales are clearly visible. Include numbers along axes only for bottom left plot of group (a 'group' is an analysis of identical markers).

☒ All plots are contour plots with outliers or pseudocolor plots.

☒ A numerical value for number of cells or percentage (with statistics) is provided.

Methodology

Sample preparation

Following incubation with guinea pig complement, beads (Thermo Fisher Scientific) were incubated with 1:100 dilution of

Sample preparation	Goat anti-Guinea Pig C3 FITC in PBS for 15 minutes at room temperature. Beads were washed three times with PBS, and then fixed with 4% paraformaldehyde for 15 minutes at room temperature. Beads were washed once with PBS, resuspended in PBS, and analyzed by flow cytometry.<br>SNAP-tagged 293F cells expressing EGFP and EBOV GP were incubated for 30 min with SNAP-Surface Alexa Fluor (AF) 647 substrate, washed twice with PBS-1% BSA, fixed with 4% methanol-free formaldehyde solution (Thermo Fisher Scientific) and kept overnight at 4℃ in dark. Next, cells were washed twice with PBS and analyzed by flow cytometry.
Instrument	LSR II; Accuri C6+ cytometer (BD Biosciences)
Software	FloJo Version X
Cell population abundance	We analyzed the specific fluorescence signals from beads/cells, which represented >95% of the events recorded.
Gating strategy	For the ADCD assay, bead population was first gated in FSC-A/SSC-A. Next, PerCP-Cy5.5 positive beads were gated (MFI>103), and analyzed for FTC signal on a histogram. For cell-based assays, cell populations were gated based on forward and side scatter cell characteristics, and next gated for EGFP, AF647, PE or PerCP-Cy5.5. The cells stained with secondary antibody only were included as the negative control.

☒ Tick this box to confirm that a figure exemplifying the gating strategy is provided in the Supplementary Information.
